# About Scarlet Fever

**Published:** 1887-09-17

**Authors:** 


### Till the Doctor Comes.

[Inquiries and Communications for this column should be addressed "Physician," care of the Editor of The Hospital.]

### XXVI.—ABOUT SCARLET FEVER.

We published on September 3rd an article on Scarlet Fever, giving an account of the most recent opinions as to its origin, with instructions concerning its home management. There is now in London a decided epidemic of this disease, and it cannot be a work of supererogation to call attention again to some particulars which ought to be familiar to every household. During epidemic periods the spread of the disease depends almost entirely upon the public and hardly at all upon doctors. In this country, too, there are very few positive laws on the subject, and to a large extent every family is free to do what is right in its own eyes.

It may be taken for granted that the community as a whole has no desire to witness the general spread of a dangerous fever. Even doctors, to whom such an occurrence may be regarded as a kind of harvest, do not desire it. On the contrary, they, knowing the danger, are much more urgent than any other class to put a stop to its ravages. One of the prime conditions of the prevention of infection is the early discovery of individual cases. It cannot be expected that fathers and mothers can be taught in a newspaper article how to diagnose a case of scarlet fever, nor is it necessary. But any parent, however inexperienced, is able to see when a child is ill; and the great point to be impressed upon people at this and all epidemic periods is that every case of illness in children should be attended to immediately. At ordinary times, if a child be a little sick, it is not necessary either to put him to bed or to send for the doctor, at any rate until some marked symptoms show themselves. But at a time like the present a very much more decided degree of promptitude is imperatively called for. The rule now should be that as soon as a child shows any signs of illness, however slight, he should either be put to bed, or, if that seem unnecessary, into a separate room. No one else should go near the room except the person who is to attend him. The room should be stripped of woollen articles, curtains, carpets, etc., in anticipation of the worst, and a sheet soaked in some disinfectant solution hung up in the doorway. If the child do not get rapidly worse, there may be no immediate necessity to send for the doctor, though where the fee is not a consideration it would be better to send for him at once.

Supposing, however, that at bedtime, or in the following morning, there are indications of increasing illness accompanied by fever, there should not be an hour's delay in seeking medical guidance. The prompt compliance with these two rules—of instant isolation and the placing of the patient in the doctor's charge—are more efficient in preventing the spread of infection than any other measures whatsoever. Scarlet fever is eminently one of those diseases which can be kept within limits if immediate steps be taken. The present writer has again and again had single cases in large families, and by the measures above indicated has succeeded in confining the disease to the original patient. It is everybody's duty, as it is everybody's interest, to spare neither trouble nor expense in stamping out with the utmost promptitude this dangerous children's scourge.

The fact that the present epidemic consists principally of mild cases probably diminishes the public alarm, and with it the public care. But it should always be remembered that what are called the *sequelæ* of scarlet fever may follow mild attacks as easily as severe ones. The principal *sequela* is inflammation of the kidneys; and to prevent this formidable result, if for no other reason, everyone should resolve to use all possible means to check the growing outbreak.

